# Molecular characterization of vancomycin-resistant *Staphylococcus aureus* strains isolated from clinical samples: A three year study in Tehran, Iran

**DOI:** 10.1371/journal.pone.0183607

**Published:** 2017-08-30

**Authors:** Marjan Shekarabi, Bahareh Hajikhani, Alireza Salimi Chirani, Maryam Fazeli, Mehdi Goudarzi

**Affiliations:** 1 Department of Microbiology, School of Medicine, Shahid Beheshti University of Medical Sciences, Tehran, Iran; 2 Microbiology Department, Faculty of Biological Sciences, Shahid Beheshti University, Tehran, Iran; 3 WHO Collaborating Center for Reference and Research on Rabies, Pasteur Institute of Iran, Tehran, Iran; Leibniz-Institute DSMZ, GERMANY

## Abstract

**Introduction:**

Emergence of vancomycin-intermediate *Staphylococcus aureus* (VISA) and vancomycin-resistant *S*. *aureus* (VRSA) strains has led to great concern in global public health in both developing and developed countries. This study investigated distribution and molecular characterization of VRSA strains in Tehran’s hospitals using a combination of molecular typing methods.

**Materials and methods:**

A total of 1789 *S*. *aureus* isolates obtained between 2014 and 2017 and were characterized using antibiogram, SCCmec typing, spa typing, and multilocus-sequence typing. Resistance to vancomycin was determined by E-test method. After confirmation of the isolated VRSA strain, genetic analysis was performed by evaluating *vanA* and *vanB* genes presence.The presence of resistance (*ermA*, *ermB*, *ermC*, *mupA*, *msrA*, *msrB*, *tetM*, *ant (4΄)-Ia*, *aac (6΄)-Ie/aph (2˝)*, *aph (3΄)-IIIa*) and toxin (*etb*, *eta*, *pvl*, *tst*) encoding genes was investigated by the polymerase chain reaction (PCR) technique.

**Results:**

Of all *S*. *aureus* tested isolates, four isolates were confirmed as VRSA isolates and two isolates confirmed as VISA isolates. ST5- SCC*mec* II/t002 and ST239-SCC*mec* III/t037 strains had MIC values of 512μg/ml, ST239-SCC*mec* III/t037 and ST8-SCC*mec*IV/t008 strains had MIC values of 64μg/ml and ST22-SCC*mec* IV/t790 and ST239-SCC*mec* III/t030 strains had MIC values ≥ 8 μg/ml. *pvl*-encoding gene was confirmed in ST8-SCC*mec*IV/t008 and ST22-SCC*mec* IV/t790 strains. The isolates differed in the carriage of resistance and toxin encoding genes.

**Conclusions:**

The study revealed the existence of VRSA strains in capital of Iran, Tehran. To our knowledge, this is the first report of ST239-SCC*mec* III/t037 as VRSA strain. These findings support the need for future surveillance studies on VRSA strains to keep the emergence and transmission of these isolates to a minimum.

## Introduction

*Staphylococcus aureus* (*S*. *aureus*), a major cause of infection in either hospitals or within communities across the world, has developed resistance to commonly prescribed antimicrobial agents [1]. The most important factor contributing to the successful extensive distribution of this nosocomial pathogen is stated to be its remarkable ability to acquire resistance to new antimicrobial agents [2]. Shortly after the introduction of penicillin as a first therapeutic option for the treatment of infections caused by penicillin-resistant *S*. *aureus*, methicillin-resistant *S*. *aureus* (MRSA) emerged in the 1960s, and since the 1980s, vancomycin has become the drug of choice for the treatment of serious MRSA infections in many healthcare institutions [3]. Although vancomycin has been the most reliable therapeutic agent against infections caused by MRSA, there has been an alarming emergence of *S*. *aureus* strains with decreased susceptibility to vancomycin and other glycopeptides [3, 4]. The first vancomycin-resistant *Enterococcus faecalis* (VRE) strain was reported in France in 1988. The acquisition of mobile genetic elements containing the *vanA* gene by *S*. *aureus* from *E*. *faecium* in laboratory mice caused great concern about this transfer in nature [5]. In 1997, the first clinical isolate of *S*. *aureus* with intermediate resistance to vancomycin was reported from Japan. Additional vancomycin-intermediate *S*. *aureus* (VISA) strains (minimum inhibitory concentration [MIC] equal to 8 μg/ml) have been isolated in several countries, including the United States [6], France [7], Brazil [8], South Korea [9], and other countries [10]. The thickened and poorly cross-linked cell wall layer of a VISA strain presents increased amounts of D-Ala-D-Ala building blocks of cell wall as a binding site for vancomycin, resulting in diminished effects of vancomycin due to competition [4]. The first vancomycin-resistant *Staphylococcus aureus* (VRSA) strain (MIC>256 μg/ml) due to the acquisition of the *vanA* was reported in 2002 from Michigan [11], and then, in the same year, in Pennsylvania and in 2004 in New York [5, 12]. A high level of resistance to vancomycin occurs due to the function of the *van* gene complex. The product of the *vanA* gene is a ligase that leads to alteration of this dipeptide residue from D-Ala-D-Ala to D-alanyl-D-lactate (D-Ala-D-Lac), a dipeptide with substantially lower affinity for the antibiotic. The *vanA* gene is not found in any VISA strain [4].

Though there are few reports of vancomycin-resistant *Staphylcoccus aureus* (VRSA) worldwide, several cases of VRSA were reported in different provinces of Iran, including Tehran, Mashhad, Isfahan, Mazandaran, Gilan and Lorestan [13]. Based on this rationale, it seems that Iran is a hotspot region for the emergence of VRSA isolates, and their incidence may be on the rise. Keeping this in view, the current study investigated the occurrence and molecular characterization of VRSA strains in Tehran, Iran, during three years.

## Material and methods

### Bacterial isolation and identification

A total of 1789 *S*. *aureus* isolates were investigated in the period of three years from March 2014 to February 2017 in five university hospitals. The strains were collected from various clinical specimens including wound, blood, pus, urine, catheters and body fluids (bronchoalveolar lavage and cerebrospinal fluid). The study protocol was approved by the Ethics Committee of Shahid Beheshti University of Medical Sciences (IR.SBMU. MSP.REC.1396.148). Identification of the clinical isolates of *S*. *aureus* was performed by traditional biochemical methods including Gram staining, growth patterns on mannitol salt agar, catalase testing, rabbit plasma coagulase testing, and DNase testing. To definitively identify positive *S*. *aureus* isolates, they were subjected to polymerase chain reaction (PCR) for *nucA* gene [14, 15].

### Detection of VRSA isolates

To screen for vancomycin resistance, all *S*. *aureus* isolates were inoculated on vancomycin agar screening medium. Vancomycin agar screen plates were prepared In-house by addition of 6 mg/L vancomycin to brain heart infusion (BHI) agar (Merck, Germany) (BHI-V6). All strains were tested for growth on BHI-V6 by spot inoculate 10 μl of each isolate with a 0.5 McFarland standard in saline onto the surface of the BHI-V6 and incubating the plate for 48 hours in ambient air at 35°C. Growth was considered as a positive result. *E*. *fecalis* ATCC 29212 was used as a vancomycin susceptible and *E*. *fecalis* ATCC 51299 was used as a reference control, in every test run [16].The isolates that had grown in vancomycin agar screening medium were further investigated to confirm the identifications [17].

### Antimicrobial susceptibility testing

The antibiotic-resistance profile was determined by the Kirby-Bauer disk diffusion technique in accordance with the Clinical and Laboratory Standards Institute (CLSI) guideline [18] using 15 antibiotic discs including penicillin (PG 10 μg), ceftriaxon (CRO 30 μg), kanamycin (K 30 μg), ciprofloxacin (CIP 5μg), rifampicin (RP 5μg), clindamycin (CD 2 μg), quinupristin-dalfopristin (SYN 15μg), tetracyclin (T 30 μg), erythromycin (E 15 μg), linezolid (LZD 30 μg), teicoplanin (TEC 30 μg), amikacin (AK 30 μg), tobramycin (TN 10 μg), gentamicin (GM 10 μg), and trimethoprim- sulfamethoxazole (TS 2.5 μg). The MRSA isolates were screened using a cefoxitin disc (30 μg) on Mueller Hinton agar plates supplemented with 4% NaCl and were confirmed by the amplification of *mec*A gene by polymerase chain reaction (PCR) method [19]. All antibiotic disks used in this research were supplied by Mast, UK. The standard reference strain *S*. *aureus* ATCC 25923 and ATCC 29213 were used as a quality control strain in every test run.

### MIC testing of *S*. *aureus* isolates

The E-test method (bioMerieux, Marcey l’Etoile, France) was used for the minimum inhibitory concentration (MIC) determination of *S*. *aureus* isolates according to the manufacturer’s instructions. The standard reference strain *S*. *aureus* ATCC 25923 and *Enterococcus faecalis* ATCC 51299 were used as vancomycin susceptible and resistant quality control strains in every test run. According to the CLSI guidelines, MIC breakpoints for vancomycin were defined as follows: susceptible, ≤2 μg/ml; intermediate, 4–8 μg/ml; and resistant, ≥16 μg/ml [18].

### Extraction of genomic DNA

DNA of strains was extracted using the commercial kit InstaGene Matrix (BioRad, Hercules co., CA, USA). According to the manufacturer’s protocol for bacterial cells, we added lysostaphin (Sigma–Aldrich co., USA) at a final concentration of 30μg/ml for cell wall lysis. After extraction, the purity of DNA was assessed using a spectrophotometer.

### Detection of resistance and toxin encoding genes

PCR was performed to determine the presence of resistance (*vanA*, *vanB*, *mecA*, *mupA*, *ermA*, *ermB*, *ermC*, *msrA*, *msrB*, *tetM*, *ant (4΄)-Ia*, *aac (6΄)-Ie/aph (2˝)*, *aph (3΄)-IIIa*) and toxin (*etb*, *eta*, *pvl*, *tst*) encoding genes. The primer sequences are presented in Table 1.

**Table 1:** Oligonucleotide primers used in this study.

Target	primer	Primer sequence (5´→3´)	Product size (bp)	Reference
*nucA*	F	GCGATTGATGGTGATACGGTT	270	[1]
	R	AGCCAAGCCTTGACGAACTAAAGC		
*mecA*	F	AGAAGATGGTATGTGGAAGTTAG	583	[17]
	R	ATGTATGTGCGATTGTATTGC		
*luk-PV*	F	TTCACTATTTGTAAAAGTGTCAGACCCACT	180	[20]
	R	TACTAATGAATTTTTTTATCGTAAGCCCTT		
*tst-1*	F	TTATCGTAAGCCCTTTGTTG	398	[17]
	R	TAAAGGTAGTTCTATTGGAGTAGG		
*eta*	F	GCAGGTGTTGATTTAGCATT	93	[2]
	R	AGATGTCCCTATTTTTGCTG		
*etb*	F	ACAAGCAAAAGAATACAGCG	226	[2]
	R	GTTTTTGGCTGCTTCTCTTG		
*ant(4΄)-Ia*	F	AATCGGTAGAAGCCCAA	135	[2]
	R	GCACCTGCCATTGCTA		
*aac(6΄)-Ie/aph(2˝)*	F	CCAAGAGCAATAAGGGCATACC	222	[2]
	R	CACACTATCATAACCACT		
*aph(3΄)-IIIa*	F	CTTGATCGAAAAATACCGCTGC	269	[2]
	R	TCATACTCTTCCGAGCAAA		
*ermA*	F	TATCTTATCGTTGAGAAGGGATT	139	[21]]
	R	CTACACTTGGCTGATGAAA		
*ermB*	F	CTATCTGATTGTTGAAGAAGCATT	141	[21]
	R	GTTTACTCTTGGTTTAGGATCAAA		
*ermC*	F	AATCGTCAATTCCTGCATGT	299	[21]
	R	TAATCGTGGAATACGGGTTTG		
*msrA*	F	GGCACAATAAGAGTG TTTAAAGG	940	[22]
	R	AAGTTATATCATGAATAGATTGTCCTGTT		
*msrB*	F	TATGATATCCATAATAATTATCCAATC	595	[22]
	R	AAGTTATATCATGAATAGATTGTCCTGTT		
*mupA*	F	CCCATGGCTTACCAGTTGA	1158	[23]
	R	CCATGGAGCACTATCCGA		
*tetM*	F	AGTGGAGCGATTACAGAA	158	[2]
	R	CATATGTCCTGGCGTGTCTA		
*vanA*	F	GGCAAGTCAGGTGAAGATG	713	[17]
	R	ATCAAGCGGTCAATCAGTTC		
*vanB*	F	GTGACAAACCGGAGGCGAGGA	430	[24]
	R	CCGCCATCCTCCTGCAAAAAA		

### Multiplex PCR amplification for SCC*mec* typing

Different SCC*mec* types were determined by specific primers are described by Boye et al [25]. SCC*mec* types were identified by comparing the banding patterns of MRSA to ATCC 10442 (SCC*mec* type I), N315 (SCC*mec* type II), 85/2082 (SCC*mec* type III), MW2 (SCC*mec* type IVa), WIS (SCC*mec* type V), as reference strains.

### *spa* typing

*Spa* typing was performed as described by Harmsen et al. [26] *spa* gene PCR products were subjected to DNA sequence analysis, and their nucleotide sequences on both strands were determined using an ABI Prism 377 automated sequencer (Applied Biosystems, Perkin-Elmer co., Foster City, CA). Sequence editing was done using Chromas software (version 1.45, Australia). Edited sequences were assigned to particular *spa* types according to the guidelines described by a Ridom SpaServer database (http://www.spaserver.ridom.de).

### Multi-locus sequence typing (MLST)

MLST with standard primers introduced by the MLST database was performed on all isolates based on seven housekeeping genes (*arc*C, *aro*E, *glp*F, *gm*K, *pta*, *tpi*A and *yqi*L) as described by Enright et al. Isolates were assigned a sequence type (ST) according to the MLST website (http://www.mlst.net/).

## Results

Of 1789 *S*. *aureus* isolates analysed, four isolates were resistant to vancomycin and were confirmed as VRSA, based on MIC results and *vanA* gene detection. All strains were isolated from hospitalized patients. Two of these isolates had MIC values of 512μg/ml, two strains had MIC values of 64μg/ml. Intermediate resistance to vancomycin was observed in two isolates with MIC ≥ 8 μg/ml. The *vanA* gene was not found in VISA strains. It is worth noting that none of isolates carried *vanB* genes. All 6 strains were methicillin-resistant and carried *mecA*. All 4 case patients infected with VRSA had received vancomycin therapy during the 11 months prior to infection while 2 cases patient infected with VISA had not had any treatment with vancomycin but had previously received glycopeptides. Interestingly, all the isolates were found to be positive for *tst* encoding gene. According to the MLST method, VRSA isolates were assigned to three different sequence types (STs) (ST5, ST239, and ST8). *spa* typing revealed that spa types t002, t037, and t008 belonged to VRSA isolates, while spa types t790 and t030 were isolated from VISA isolates. None of the isolates carry all the resistance encoding genes simultaneously. All VRSA and VISA isolates were MDR. Surprisingly, ST8-SCC*mec*IV/t008 isolate with vancomycin MIC 64 μg/ml does not carry the *vanA* gene. Molecular characterization of VRSA and VISA strains are described in Table 2.

**Table 2:** Molecular characterization of VRSA and VISA strains.

Molecular types	Sample/ward	Phenotypic resistance	Genotypic resistance	Toxin profile	Vancomycin MIC (mg/ml)	MIC Interpretive Breakpoints R/I/S^a^ μg/mL		
						≤ 2	4–8	≥16
ST5- SCC*mec* II/t002	Throat/ICU	PG,CRO,K,CIP,CD,T,E,TEC,AK,TN,GM,TS	*vanA*, *mecA*, *ermA*, *ermB*, *msrA*, *tetM*, *ant (4΄)-Ia*, *aac (6΄)-Ie/aph (2˝)*, *aph (3΄)-IIIa*	tst, eta, etb	512	Resistant		
ST239-SCC*mec* III/t037	bronchial aspirate/Internal	PG,CRO,K,CIP,RP,CD,T,E,TN,GM	*vanA*, *mecA*, *ermA*, *ermB*, *msrA*, *tetM*, *ant (4΄)-Ia*, *aph (3΄)-IIIa*	tst	512	Resistant		
ST239-SCC*mec* III/t037	Wound/ICU	PG,CRO,K,CIP,RP,CD,T,E,AK,TN,GM,TS	*vanA*, *mecA*, *ermA*, *ermB*, *ermC*, *msrB*, *tetM*, *ant (4΄)-Ia*,	tst	64	Resistant		
ST8-SCC*mec*IV/t008	Blood/Oncology	PG,CRO,K,CIP,RP,CD,T,E,AK,TN,TS	*mecA*, *tetM*, *ant (4΄)-Ia*, *aac (6΄)-Ie/aph (2˝)*, *aph (3΄)-IIIa*	pvl, tst	64	Resistant		
ST22-SCC*mec* IV/t790	Blood/ICU	PG,CRO,CD,T,E,SYN,AK,TN,GM,TS	*mecA*, *ermA*, *ermB*, *ermC*, *msrA*, *msrB*, *tetM*, *ant (4΄)-Ia*, *aac (6΄)-Ie/aph (2˝)*, *aph (3΄)-IIIa*	pvl,tst, eta, etb	8	Intermediate Resistant		
ST239-SCC*mec* III/t030	Catheter/Infectious	PG,CRO,K,CD,T,E,AK,TN,GM,TS	*mecA*, *ermA*, *ermB*, *ermC*, *msrA*, *msrB*, *tetM*, *ant (4΄)-Ia*, *aac (6΄)-Ie/aph (2˝)*, *aph (3΄)-IIIa*	tst	8	Intermediate Resistant		

PG: penicillin, CRO: ceftriaxone, K: kanamycin, CIP: ciprofloxacin, RP: rifampicin, CD: clindamycin, SYN: quinupristin-dalfopristin, T: tetracyclin, E: erythromycin, LZD: linezolid, TEC: teicoplanin, AK: amikacin, TN: tobramycin, GM: gentamicin, TS: trimethoprim- sulfamethoxazole.

^a^ MIC breakpoints applied were those recommended for *S*. *aureus* by the Clinical and Laboratory Standards Institute (CLSI), susceptible, ≤2 μg/ml; intermediate, susceptible, ≤2 μg/ml; intermediate, 4–8 μg/ml; and resistant, ≥16 μg/ml; and resistant, ≥16 μg/ml [18].

## Discussion

In the past few years, several antibiotics have been noted to be less effective in the treatment of S. aureus infections, leading to treatment failure. In these contexts, VRSA and VISA strains are highly prominent. Isolates of vancomycin resistant *S*. *aureus* have emerged in many parts of the world, such as the United States [11, 12], Japan [27], India [28, 29], and Iran [30, 31]. Although VRSA strains have hitherto been thought to be rare, the current findings on the prevalence of VRSA strains in Iran have demonstrated a steadily increased rate of VRSA isolates. In this study, we found an MRSA strain with high levels of resistance to vancomycin (MIC 512μg/ml) isolated from patient who had serious respiratory tract infection with long-time hospitalization and previous use of vancomycin. Our data showed that this isolate belonged to ST5-SCC*mec* II/t002 clone. Most of the previously isolated VRSA strains in the United States belong to sequence type 5 (ST5), one of the widespread MRSA clones [32]. It is noteworthy that other genetic and phenotypic backgrounds of our isolate were similar to those of its US counterpart, suggesting that the Iranian isolate has a strong relationship with the ST5 US strain. Several studies have reported variable resistance and virulence markers in ST5 strains [33]. In the present study, ST5-SCC*mec* II/t002 clone was positive for *eta*, *etb* and *tst* genes and carry aminoglycoside, macrolide and tetracycline resistance genes. According to the antibiotic susceptibility testing, this strain was resistant to most of the tested antibiotics except rifampicin, quinupristin-dalfopristin and linezolid, which is in contrast to previous studies that reported susceptible VRSA isolates to many of the antibiotics [12, 34, 35].

It is documented that ST239 is divided into three clades—the South American, European, and Asian clades. Furthermore, multi-resistant ST239 and positive *tst* isolates are much more frequent in the USA, Europe, and some Asian countries [36]. Our data showed that ST239-SCC*mec* III/t037 VRSA strains were PVL-negative, but was positive for *tst* gene in addition to genes, *vanA*, *mecA*, *ermA*, *ermB*, *ermC*, *tetM*, *ant (4΄)-Ia*, *aph (3΄)-IIIa*. These strains were significantly resistant to many antibiotic groups. This finding is in contrast to the results of a study in Iran by Havaei et al. [37], who reported ST239-SCC*mec* III/t037 as a VISA strain with MIC 4 μg/ml, which was obtained from the blood sample of a 50-year-old man. ST239 as a VISA strain was previously reported from Pennsylvania [38] and New Zealand [39]. Also, a study conducted in Iran by Azimian et al. [17] reported one VRSA isolate separated from the bronchial aspirate of a 26-year-old man that belonged to ST1283-SCC*mec* III/t037, a single locus variant of ST239 (an endemic clone in most Asian countries except South Korea and Japan). To the best of our knowledge, this is the first report of ST239-SCC*mec* III/t037 as VRSA strain. Literature have shown that a single point mutation in position 483 of the guanylate kinase gene (*gmk*) with the replacement of thymine by guanine (T → G), in ST239 can lead to the generation of ST1283. ST239, as previously reported, is a strain with a high level of resistance to vancomycin in Iran that should be more considered [17].

In contrast to previously published data from Iran, which reported ST8-t008 isolate as a VISA strain [37], in this study we found ST8-SCC*mec*IV/t008 isolate with vancomycin MIC of 64μg/ml that was isolated from the blood sample of a 34-year-old man. The result of the present study shows a significant rise in the resistance of vancomycin in ST8 isolate in Iran. In line with Tiwari et al.’s study in 2006 from India [40], ST8-SCC*mec*IV/t008 isolate was noted to be a VRSA *van* gene-negative strain. The absence of *vanA* gene in the present isolates does not rule out that these strains are not VRSA. It is thought that cell wall thickening may be responsible for the development of vancomycin resistance in these isolates. Although antimicrobial resistance pattern in ST8-SCC*mec*IV/t008 isolates may vary, trimethoprim- sulfamethoxazole resistance in ST8 isolates is reported by several investigators [41], which is in accordance with the present study. This is believed to be the first report of ST8-VRSA from Tehran, Iran, as well as Asia, and may soon become a global problem. This emergence of ST8-SCC*mec*IV/t008 VRSA/VISA may be attributed to the building of selective pressure of vancomycin as the main antimicrobial agent available to treat life-threatening infections with MRSA.

VISA strains with a heterogeneous genetic background were reported in several studies from Iran [1], India [40], US [38], Bangladesh [42], New Zealand [39] and China [43]. There is not enough information about the prevalence rate of VISA strains in Iran; however, in 2005, Saderi et al. reported the prevalence rate of 1.8% for VISA isolated from clinical samples based on the evaluation of four teaching hospitals in Tehran [44]. Havaei et al. reported five VISA strains (2.9%) amongst 171 *S*. *aureus* isolated from different Iranian hospitals [37]. Based on literature, there is an upward trend in the prevalence of VISA strains in Iran [1, 37]. In the present survey, two isolates with intermediate-resistance to vancomycin were related to the ST22-SCC*mec* IV/t790 and ST239-SCC*mec* III/t030 clones. Our result of VISA genotyping was the same as the previous report by Goudarzi et al. [1] from Iran, who reported two VISA isolates with genetically characteristic SCC*mec* type IV, *spa* type t790, and ST22.

In this study, it was shown that other VISA isolates belonged to ST239-SCC*mec* III/t030 clone, which is in accordance with Bozdogan et al.’s study, in which intermediate resistance to vancomycin in MRSA ST239 clone was reported [38]. ST239 VISA was also previously identified in Iran [37] and New Zealand [39]. The current finding on the prevalence of VRSA strains further demonstrates the necessity to implement infection-control precautions to prevent the spread of resistance to vancomycin in our hospitals.

## Conclusion

To summarize, the results of this study showed that VRSA and VIS isolated in belonged to diverse genetic backgrounds which should be seriously taken into consideration. Although we confined our study within Tehran, the emergence of VRSA and VISA strains might also be prevalent in other provinces of Iran. Based on our antimicrobial susceptibility testing results, linezolid may be the most effective drug against VRSA strains. It seems that performing continuous and nationwide surveillance programs to map the vancomycin susceptibility pattern in our country is necessary.

## Supporting information

S1 File Dataset. (RAR)

## References

[1] Goudarzi M, Goudarzi H, Figueiredo AMS, Udo EE, Fazeli M, Asadzadeh M, et al Molecular characterization of methicillin resistant Staphylococcus aureus strains isolated from intensive care units in Iran: ST22-SCCmec IV/t790 emerges as the major clone. PloS one. 2016;11(5):e0155529 doi: 10.1371/journal.pone.0155529 27171373 10.1371/journal.pone.0155529 PMC4865093

[2] Nezhad RR, Meybodi SM, Rezaee R, Goudarzi M, Fazeli M. Molecular Characterization and Resistance Profile of Methicillin Resistant Staphylococcus aureus Strains Isolated from Hospitalized Patients in Intensive Care Unit, Tehran-Iran. Jundishapur J Microbiol. 2017;10(3)): e41666.

[3] Appelbaum P. The emergence of vancomycin‐intermediate and vancomycin‐resistant Staphylococcus aureus. Clin Microbiol Infect. 2006;12(s1):16–23. 10.1111/j.1469-0691.2006.01344.x 16445720

[4] Gardete S, Tomasz A. Mechanisms of vancomycin resistance in Staphylococcus aureus. J Clin Invest. 2014;124(7):2836 doi: 10.1172/JCI68834 24983424 10.1172/JCI68834 PMC4071404

[5] Sievert DM, Rudrik JT, Patel JB, McDonald LC, Wilkins MJ, Hageman JC. Vancomycin-resistant Staphylococcus aureus in the United States, 2002–2006. Clin Infect Dis. 2008;46(5):668–74. doi: 10.1086/527392 18257700 10.1086/527392

[6] Walsh TR, Howe RA. The prevalence and mechanisms of vancomycin resistance in Staphylococcus aureus. Annu Rev Microbiol. 2002;56(1):657–75. 12142482 10.1146/annurev.micro.56.012302.160806

[7] Ploy M, Grelaud C, Martin C, De Lumley L, Denis F. First clinical isolate of vancomycin-intermediate Staphylococcus aureus in a French hospital. Lancet. 1998;351(9110):1212. 10.1016/s0140-6736(05)79166-2 9643727

[8] Oliveira GA, Dell'Aquila AM, Masiero RL, Levy CE, Gomes MS, Cui L, et al Isolation in Brazil of nosocomial Staphylococcus aureus with reduced susceptibility to vancomycin. Infect Control Hosp Epidemiol. 2001;22(7):443–8. doi: 10.1086/501932 11583214 10.1086/501932

[9] Kim M-N, Pai CH, Woo JH, Ryu JS, Hiramatsu K. Vancomycin-intermediate Staphylococcus aureus in Korea. J Clin Microbiol. 2000;38(10):3879–81. 11015427 10.1128/jcm.38.10.3879-3881.2000 PMC87500

[10] Weinstein RA, Fridkin SK. Vancomycin-intermediate and-resistant Staphylococcus aureus: what the infectious disease specialist needs to know. Clin Infect Dis. 2001;32(1):108–15. doi: 10.1086/317542 11118389 10.1086/317542

[11] Chang S, Sievert DM, Hageman JC, Boulton ML, Tenover FC, Downes FP, et al Infection with vancomycin-resistant Staphylococcus aureus containing the vanA resistance gene. N Engl J Med. 2003;348(14):1342–7. doi: 10.1056/NEJMoa025025 12672861 10.1056/NEJMoa025025

[12] Control CfD, Prevention. Vancomycin-resistant Staphylococcus aureus—Pennsylvania, 2002. MMWR Morbidity and mortality weekly report. 2002;51(40):902 12418544

[13] Askari E, Zarifian A, Pourmand M, Naderi-Nasab M. High-level vancomycin-resistant Staphylococcus aureus (VRSA) in Iran: A systematic review. J Med Bacteriol. 2015;1(3–4):53–61.

[14] Goudarzi M, Seyedjavadi SS, Nasiri MJ, Goudarzi H, Nia RS, Dabiri H. Molecular characteristics of methicillin-resistant Staphylococcus aureus (MRSA) strains isolated from patients with bacteremia based on MLST, SCCmec, spa, and agr locus types analysis. Microb Pathog. 2017;104:328–35. doi: 10.1016/j.micpath.2017.01.055 28159661 10.1016/j.micpath.2017.01.055

[15] Goudarzi M, Bahramian M, Tabrizi MS, Udo EE, Figueiredo AMS, Fazeli M, et al Genetic diversity of methicillin resistant Staphylococcus aureus strains isolated from burn patients in Iran: ST239-SCCmec III/t037 emerges as the major clone. Microb Pathog. 2017;105:1–7. doi: 10.1016/j.micpath.2017.02.004 28179118 10.1016/j.micpath.2017.02.004

[16] Kosowska-Shick K, Ednie LM, McGhee P, Smith K, Todd CD, Wehler A, et al Incidence and characteristics of vancomycin nonsusceptible strains of methicillin-resistant Staphylococcus aureus at Hershey Medical Center. Antimicrob Agents Chemother. 2008;52(12):4510–3. doi: 10.1128/AAC.01073-08 18838583 10.1128/AAC.01073-08 PMC2592881

[17] Azimian A, Havaei SA, Fazeli H, Naderi M, Ghazvini K, Samiee SM, et al Genetic characterization of a vancomycin-resistant Staphylococcus aureus isolate from the respiratory tract of a patient in a university hospital in northeastern Iran. J Clin Microbiol. 2012;50(11):3581–5. doi: 10.1128/JCM.01727-12 22933598 10.1128/JCM.01727-12 PMC3486207

[18] Clinical and Laboratory Standards Institute. Performance standards for antimicrobial susceptibility testing; Twenty-Second Informational Supplement; 2014, 31:M100-S22.

[19] Goudarzi M, Fazeli M, Goudarzi H, Azad M, Seyedjavadi SS. Spa typing of Staphylococcus aureus strains isolated from clinical specimens of patients with nosocomial infections in Tehran, Iran. Jundishapur J Microbiol. 2016;9(7):e35685 doi: 10.5812/jjm.35685 27679706 10.5812/jjm.35685 PMC5035396

[20] Jarraud S, Mougel C, Thioulouse J, Lina G, Meugnier H, Forey F, et al Relationships between Staphylococcus aureus genetic background, virulence factors, agr groups (alleles), and human disease. Infect Immun. 2002;70(2):631–41. doi: 10.1128/IAI.70.2.631-641.2002 11796592 10.1128/IAI.70.2.631-641.2002 PMC127674

[21] Martineau F, Picard FJ, Lansac N, Ménard C, Roy PH, Ouellette M, et al Correlation between the Resistance Genotype Determined by Multiplex PCR Assays and the Antibiotic Susceptibility Patterns of Staphylococcus aureus andStaphylococcus epidermidis. Antimicrob Agents Chemother. 2000;44(2):231–8. 10639342 10.1128/aac.44.2.231-238.2000 PMC89663

[22] Lina G, Quaglia A, Reverdy M-E, Leclercq R, Vandenesch F, Etienne J. Distribution of genes encoding resistance to macrolides, lincosamides, and streptogramins among staphylococci. Antimicrob Agents Chemother. 1999;43(5):1062–6. 10223914 10.1128/aac.43.5.1062 PMC89111

[23] Udo E, Jacob L, Mathew B. Genetic analysis of methicillin-resistant Staphylococcus aureus expressing high-and low-level mupirocin resistance. J Med Microbiol. 2001;50(10):909–15. doi: 10.1099/0022-1317-50-10-909 11599741 10.1099/0022-1317-50-10-909

[24] Saadat S, Solhjoo K, Norooz-Nejad M-J, Kazemi A. VanA and vanB positive vancomycin-resistant Staphylococcus aureus among clinical isolates in Shiraz, South of Iran. Oman Med J. 2014;29(5):335 doi: 10.5001/omj.2014.90 25337309 10.5001/omj.2014.90 PMC4202220

[25] Boye K, Bartels MD, Andersen IS, Moeller JA, Westh H. A new multiplex PCR for easy screening of methicillin‐resistant Staphylococcus aureus SCCmec types I–V. Clin Microbiol Infect. 2007;13(7):725–7. doi: 10.1111/j.1469-0691.2007.01720.x 17403127 10.1111/j.1469-0691.2007.01720.x

[26] Harmsen D, Claus H, Witte W, Rothgänger J, Claus H, Turnwald D, et al Typing of methicillin-resistant Staphylococcus aureus in a university hospital setting by using novel software for spa repeat determination and database management. J Clin Microbiol. 2003;41(12):5442–8. doi: 10.1128/JCM.41.12.5442-5448.2003 14662923 10.1128/JCM.41.12.5442-5448.2003 PMC309029

[27] Hiramatsu K. Vancomycin-resistant Staphylococcus aureus: a new model of antibiotic resistance. Lancet Infect Dis. 2001;1(3):147–55. doi: 10.1016/S1473-3099(01)00091-3 11871491 10.1016/S1473-3099(01)00091-3

[28] Saha B, Singh AK, Ghosh A, Bal M. Identification and characterization of a vancomycin-resistant Staphylococcus aureus isolated from Kolkata (South Asia). J Med Microbiol. 2008;57(1):72–9. 18065670 10.1099/jmm.0.47144-0

[29] Thati V, Shivannavar CT, Gaddad SM. Vancomycin resistance among methicillin resistant Staphylococcus aureus isolates from intensive care units of tertiary care hospitals in Hyderabad. Indian J Med Res. 2011;134(5):704 doi: 10.4103/0971-5916.91001 22199111 10.4103/0971-5916.91001 PMC3249970

[30] Dezfulian A, Aslani MM, Oskoui M, Farrokh P, Azimirad M, Dabiri H, et al Identification and characterization of a high vancomycin-resistant Staphylococcus aureus harboring VanA gene cluster isolated from diabetic foot ulcer. Iran J Basic Med Sci. 2012;15(2):803 23495359 PMC3586877

[31] Aligholi M, Emaneini M, Jabalameli F, Shahsavan S, Dabiri H, Sedaght H. Emergence of high-level vancomycin-resistant Staphylococcus aureus in the Imam Khomeini Hospital in Tehran. Med Princ Pract. 2008;17(5):432–4. doi: 10.1159/000141513 18685289 10.1159/000141513

[32] Zhu W, Clark NC, McDougal LK, Hageman J, McDonald LC, Patel JB. Vancomycin-resistant Staphylococcus aureus isolates associated with Inc18-like vanA plasmids in Michigan. Antimicrob Agents Chemother. 2008;52(2):452–7. doi: 10.1128/AAC.00908-07 18056272 10.1128/AAC.00908-07 PMC2224762

[33] Ranjbary AG. Isolation of vancomycin-resistant Staphylococcus aureus in a patient with burn injury. Rev Med Microbiol. 2016;27(1):36–8.

[34] Bush K. Vancomycin-resistant Staphylococcus aureus in the clinic: not quite Armageddon. Clin Infect Dis. 2004;38(8):1056–7. doi: 10.1086/382366 15095206 10.1086/382366

[35] Control CfD, Prevention. Vancomycin-resistant Staphylococcus aureus—New York, 2004. MMWR Morbidity and mortality weekly report. 2004;53(15):322 15103297

[36] Monecke S, Coombs G, Shore AC, Coleman DC, Akpaka P, Borg M, et al A field guide to pandemic, epidemic and sporadic clones of methicillin-resistant Staphylococcus aureus. PloS one. 2011;6(4):e17936 doi: 10.1371/journal.pone.0017936 21494333 10.1371/journal.pone.0017936 PMC3071808

[37] Havaei SA, Azimian A, Fazeli H, Naderi M, Ghazvini K, Samiee SM, et al Genetic characterization of methicillin resistant and sensitive, vancomycin intermediate Staphylococcus aureus strains isolated from different Iranian Hospitals. ISRN Microbiol. 2012;2012. 10.5402/2012/215275 PMC3664199 23762750

[38] Bozdogan B, Ednie L, Credito K, Kosowska K, Appelbaum PC. Derivatives of a vancomycin-resistant Staphylococcus aureus strain isolated at Hershey Medical Center. Antimicrob Agents Chemother. 2004;48(12):4762–5. doi: 10.1128/AAC.48.12.4762-4765.2004 15561854 10.1128/AAC.48.12.4762-4765.2004 PMC529230

[39] Howden BP, Seemann T, Harrison PF, McEvoy CR, Stanton J-AL, Rand CJ, et al Complete genome sequence of Staphylococcus aureus strain JKD6008, an ST239 clone of methicillin-resistant Staphylococcus aureus with intermediate-level vancomycin resistance. J Bacteriol. 2010;192(21):5848–9. doi: 10.1128/JB.00951-10 20802046 10.1128/JB.00951-10 PMC2953705

[40] Tiwari HK, Sen MR. Emergence of vancomycin resistant Staphylococcus aureus (VRSA) from a tertiary care hospital from northern part of India. BMC Infect Dis. 2006;6(1):156. 17067393 10.1186/1471-2334-6-156 PMC1634751

[41] McDougal LK, Fosheim GE, Nicholson A, Bulens SN, Limbago BM, Shearer JE, et al Emergence of resistance among USA300 methicillin-resistant Staphylococcus aureus isolates causing invasive disease in the United States. Antimicrob Agents Chemother. 2010;54(9):3804–11. doi: 10.1128/AAC.00351-10 20585117 10.1128/AAC.00351-10 PMC2934979

[42] Hasan R, Acharjee M, Noor R. Prevalence of vancomycin resistant Staphylococcus aureus (VRSA) in methicillin resistant S. aureus (MRSA) strains isolated from burn wound infections. Tzu Chi Med J. 2016;28(2):49–53. 10.1016/j.tcmj.2016.03.002 PMC5442891 28757721

[43] Zhu X, Liu C, Gao S, Lu Y, Chen Z, Sun Z. Vancomycin intermediate-resistant Staphylococcus aureus (VISA) isolated from a patient who never received vancomycin treatment. Int J Infect Dis. 2015;33:185–90. doi: 10.1016/j.ijid.2014.12.038 25543098 10.1016/j.ijid.2014.12.038

[44] Shahrbanooie R. Vancomycin resistance among clinical isolates of Staphylococcus aureus. Arch Iran Med. 2005;8(2):100–3.

